# Network Dynamics Underlying Speed-Accuracy Trade-Offs in Response to Errors

**DOI:** 10.1371/journal.pone.0073692

**Published:** 2013-09-12

**Authors:** Yigal Agam, Caitlin Carey, Jason J. S. Barton, Kara A. Dyckman, Adrian K. C. Lee, Mark Vangel, Dara S. Manoach

**Affiliations:** 1 Department of Psychiatry, Massachusetts General Hospital, Harvard Medical School, Boston, Massachusetts, United States of America; 2 Athinoula A. Martinos Center for Biomedical Imaging, Charlestown, Massachusetts, United States of America; 3 Department of Psychology, Harvard University, Cambridge, Massachusetts, United States of America; 4 Departments of Neurology, Ophthalmology and Visual Sciences, University of British Columbia, Vancouver, British Columbia, Canada; 5 Department of Psychology, University of Georgia, Athens, Georgia, United States of America; 6 Department of Speech and Hearing Sciences, University of Washington, Seattle, Washington, United States of America; University of Gent, Belgium

## Abstract

The ability to dynamically and rapidly adjust task performance based on its outcome is fundamental to adaptive, flexible behavior. Over trials of a task, responses speed up until an error is committed and after the error responses slow down. These dynamic adjustments serve to optimize performance and are well-described by the speed-accuracy trade-off (SATO) function. We hypothesized that SATOs based on outcomes reflect reciprocal changes in the allocation of attention between the internal milieu and the task-at-hand, as indexed by reciprocal changes in activity between the default and dorsal attention brain networks. We tested this hypothesis using functional MRI to examine the pattern of network activation over a series of trials surrounding and including an error. We further hypothesized that these reciprocal changes in network activity are coordinated by the posterior cingulate cortex (PCC) and would rely on the structural integrity of its white matter connections. Using diffusion tensor imaging, we examined whether fractional anisotropy of the posterior cingulum bundle correlated with the magnitude of reciprocal changes in network activation around errors. As expected, reaction time (RT) in trials surrounding errors was consistent with predictions from the SATO function. Activation in the default network was: *(i)* inversely correlated with RT, *(ii)* greater on trials before than after an error and *(iii)* maximal at the error. In contrast, activation in the right intraparietal sulcus of the dorsal attention network was *(i)* positively correlated with RT and showed the opposite pattern: *(ii)* less activation before than after an error and *(iii)* the least activation on the error. Greater integrity of the posterior cingulum bundle was associated with greater reciprocity in network activation around errors. These findings suggest that dynamic changes in attention to the internal versus external milieu in response to errors underlie SATOs in RT and are mediated by the PCC.

## Introduction

**Figure 1:**
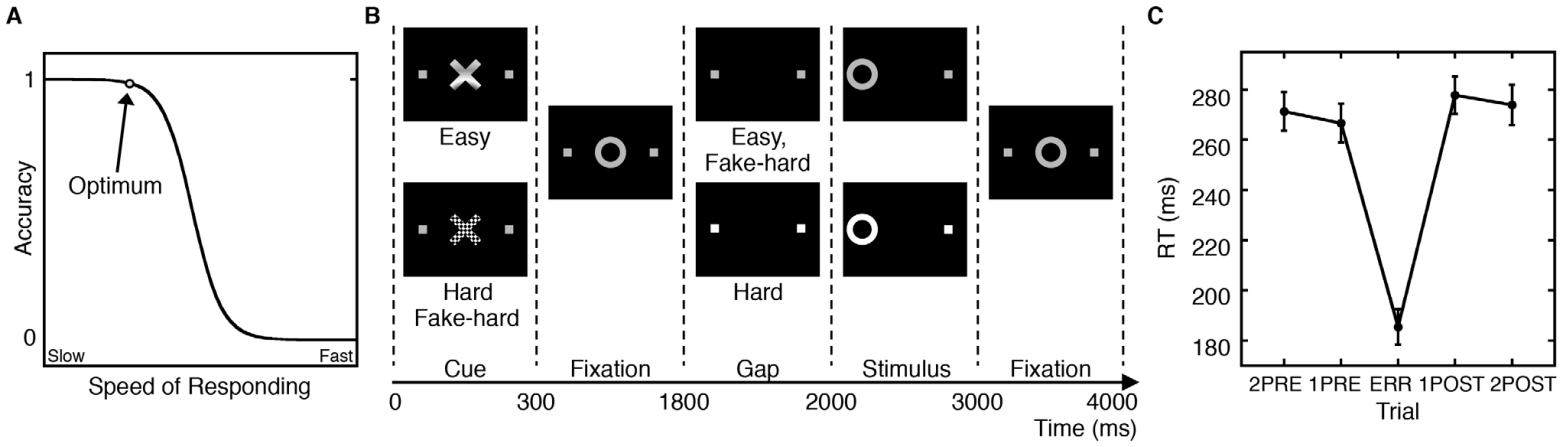
Speed-accuracy trade-offs during the antisaccade task. A: A schematic depiction of the speed-accuracy trade-off (SATO). As reaction time (RT) increases, the probability of an error decreases. The circle denotes the point where optimal accuracy is achieved with the fastest RT. Beyond this point, making faster responses entails a cost in reduced accuracy. B: The antisaccade paradigm. Schematic and timeline of the three conditions: easy, hard, and fake-hard. Each trial lasted four seconds and began with an instructional cue (300 ms), either a blue or yellow ‘X’ that indicated whether the trial was hard or easy. The mapping of cue color to trial type was counterbalanced across participants. The cue was horizontally flanked by two white squares of 0.4° width that marked the potential locations of stimulus appearance, 10° left and right of center. The squares remained visible for the duration of each run. At 300 ms, the instructional cue was replaced by a white fixation ring of 1.3° diameter at the center of the screen. At 1800 ms, the fixation ring disappeared (200 ms gap). At 2000 ms, the fixation ring reappeared at one of the two stimulus locations, right or left with equal probability. This was the imperative stimulus to which the participant responded by making a saccade in the opposite direction. The ring remained in the peripheral location for 1000 ms and then returned to the center, where participants were instructed to return their gaze for 1000 ms before the start of the next trial. Hard trials were distinguished by a 3 db increase in luminance of the peripheral squares starting during the gap. Except for the hard cue, fake-hard trials were identical to easy trials. Fixation epochs were simply a continuation of the fixation display that constitutes the final second of the previous saccadic trial. C: Mean saccadic RT as a function of trial position in relation to an error (ERR) with standard error bars.

Trial-by-trial variability in reaction time (RT) is ubiquitous in human behavior, yet its neural basis is poorly understood. In experimental studies RT variability is often modeled as random noise. While it may include a stochastic component, RT variability also reflects dynamic adjustments based on contextual factors such as whether or not the prior task was the same, performance history, and current contingencies [1], [2]. Here, we studied the neural basis of speed-accuracy trade-offs (SATOs) in response to errors. SATOs refer to trial-by-trial adjustments of RT that optimize performance. Such dynamic modulations of performance are fundamental to adaptive, flexible behavior. The SATO function depicts the non-linear relation between speed and accuracy such that faster responding does not affect accuracy, but only up to a point. Beyond that point speed and accuracy are inversely related, with slower responses having a greater probability of being correct (Fig. 1A). That transition point can be regarded as an optimum, where the best accuracy is achieved at the fastest possible speed. SATOs are generally studied by manipulating participant instructions to emphasize either the speed or accuracy of responding. These manipulations are thought to lead to strategic changes in decisions about how much evidence is required to initiate a response. In the present study, rather than manipulating instructions, we examined SATOs in response to behavioral outcomes, specifically errors, which we hypothesize primarily reflect changes in task-directed attention. Over trials, responses speed up until an error is committed i.e., pre-error speeding; [3], [4] and following an error, RT slows [i.e., post-error slowing; 5], and the probability of an error decreases. This pattern can be interpreted as a progression to riskier positions on the SATO function culminating in an error, which is followed by a shift back to safer, more careful responding with a greater likelihood of a correct response.

In the present study, we used functional MRI (fMRI) to test the hypothesis that SATOs in RT in response to errors are mediated by dynamic and reciprocal exchanges in the allocation of cognitive processing resources between the default and dorsal attention networks of the brain. Based on evidence that errors are characterized by transient declines in preparatory task-related attention [6] and/or failure to suppress neural activity that interferes with performance [7], [8] and that attention is re-oriented in correct trials that follow errors [6], we expected activation in the default and dorsal attention networks to show reciprocal patterns of activation on trials surrounding an error, and to correlate in opposite directions with changes in RT. We used diffusion tensor imaging (DTI) to test the hypothesis that these network interactions rely on the integrity of the structural connections of the posterior cingulate cortex (PCC), which, as a cortical hub with a critical role in error processing, is in a privileged position to mediate network interactions around errors (evidence reviewed below). We expected greater coordination (i.e., reciprocity) between these networks and more pronounced SATOs in RT to correlate with greater microstructural integrity of the posterior cingulum bundle.

The default network is a set of functionally and structurally connected regions [9], [10] that typically show increased fMRI activation when not engaged in an overt task and deactivation during effortful task performance [11]. The core regions of the human default network are the ventromedial prefrontal cortex, posterior cingulate/retrosplenial cortex, inferior parietal lobule, lateral temporal cortex, dorsal medial prefrontal cortex, and hippocampal formation [11]. The default network is thought to be involved in self-referential and affective processing, and ‘task-induced deactivation’ is presumed to reflect the re-allocation of limited capacity cognitive processing resources away from the internal milieu to allow greater attention to the task-at-hand [12]. Consistent with this theory, activation in the default network inversely correlates with activation in the dorsal attention network [13], [14], which typically increases activity during cognitive tasks and is involved in effortful attention [15]. During trials immediately preceding errors [8], [16] and error trials [7] there is a relative failure of task-induced deactivation, suggesting that increasing interference from internally directed thought culminates in an error. Trials that follow errors, in contrast, show relatively increased task-induced deactivation and a corresponding increase in dorsal attention network activation [8], suggesting a shift in focus from the internal milieu back to the overt task. This cyclical pattern of network activation and deactivation around error trials is likely to be reflected in behavior. This is consistent with findings that the relative connectivity [17] and activation [18] of the default and dorsal attention networks correlate with RT. Building on this literature, we hypothesized that reciprocal changes in resource allocation between the default and dorsal attention networks around errors underlie SATOs in RT and are mediated by the PCC.

The PCC is the most densely connected cortical region, both structurally [19] and functionally [20], [21] putting it in a privileged position to mediate network interactions. It is a major node of the default network and is negatively functionally connected to regions of the dorsal attention network. In the specific PCC subregions that show task-induced deactivation, functional connectivity with dorsal attention network regions is modulated by task difficulty, consistent with a role in regulating the balance between internally- and externally-directed attention that determines task-engagement [22], [23]. The PCC is also the putative generator of the error-related negativity (an event-related potential that indexes error detection) and the microstructural integrity of the posterior cingulum bundle predicts the speed of error self-correction [24]. This suggests that the PCC detects errors and through its connections with other regions including the anterior cingulate and lateral prefrontal cortex [25], initiates immediate corrective action [24]. Based on these findings that the PCC component of the default network is functionally connected with the dorsal attention network, that the strength of these connections is modulated by task difficulty, that the PCC detects errors and that the integrity of its white matter correlates with the speed of corrective action, we hypothesized that the PCC might also mediate network interactions around errors to support RT adjustments. Specifically, we hypothesized that the PCC mediates reciprocal changes in the balance of activity between the default and dorsal attention networks in response to errors. This would serve to reduce internal focus, increase attention to the task, and thereby support slower more careful responding to prevent error recurrence in subsequent trials.

To test our hypotheses that dynamic exchanges in resource allocation between default and dorsal attention networks underlie SATOs in response to errors and are mediated by the PCC we used an antisaccade task (Fig. 1B), rapid-presentation event-related fMRI, and DTI. Antisaccades require inhibition of the prepotent response of looking towards a suddenly appearing stimulus and the substitution of a gaze in the opposite direction. Antisaccades are characterized by RT adjustments consistent with the SATO [26] and errors (i.e., looking toward the stimulus) are associated with a failure of task-induced deactivation [7]. We examined the correlations of task-related activation with RT over a series of five trials at different positions relative to an error: two trials pre-error (2PRE), one trial pre-error (1PRE), error (ERR), one trial post-error (1POST), two trials post-error (2POST). In the default network, we expected relatively increased activation on pre-error trials and a corresponding speeding of RT, followed by the fastest RT and greatest activation on the error trial, consistent with findings that antisaccade errors are faster than correct responses and are associated with greater activation in the default network [7], [26]. Following the error we expected a reinstatement of task-induced deactivation and a corresponding slowing of RT. In the dorsal attention network, we expected the opposite pattern (i.e., relatively decreased activation prior to and including the error followed by a post-error increase in activation) and positive correlations of activation with RT. This prediction is consistent with findings from both monkey single-unit recording and human neuroimaging studies of antisaccades suggesting that greater inhibition of the frontal eye field [reflected in reduced firing and increased fMRI signal, 27] is associated with lower error rates and slower correct responses [1], [28], [29]. This pattern of network activation and RT changes would suggest that reciprocal changes in activation between default and dorsal attention networks underlie SATOs and reflect changes in the allocation of attention from the internal milieu to the task-at-hand. We also examined whether an index of coordination between these networks correlated with SATOs in RT. Finally, to test whether network coordination and SATOs rely on the structural connectivity of the PCC, we examined the relations of fractional anistropy (FA, a measure of white matter microstructrual integrity) of the posterior cingulum bundle with both the index of network coordination and post-error slowing. We expected greater integrity of the posterior cingulum bundle to be associated with greater coordination between the default and dorsal attention networks (i.e., more pronounced reciprocal pattern of activation) and more pronounced post-error slowing. In summary, we used fMRI and DTI to test the hypotheses that dynamic changes in attention to the internal versus external milieu in response to errors underlie SATOs in RT and are mediated by the PCC.

## Methods

### Participants

Of the 46 healthy participants enrolled in the study, 35 (14 female; age 35±13 years) met the criterion for inclusion of having a minimum of ten sequences of trials in which an error (ERR) was preceded by two correct trials (2PRE and 1PRE) and followed by 2 correct trials (1POST, 2POST). The error rate for all enrolled participants (n = 46) was 14±12% (mean±SD). After exclusion, the remaining 35 participants had an error rate of 17±11%. All participants gave written informed consent, and the protocol was approved by the Partners Human Research Committee.

### Antisaccade Paradigm

The antisaccade paradigm was programmed in Matlab Psychtoolbox (Mathworks, Natick, MA). It consisted of a pseudorandom sequence of three antisaccade conditions and a fixation condition lasting 2, 4, or 6 s (Fig. 1B). The three antisaccade conditions were: hard (40%), easy (50%), and fake-hard (10%). Hard trials introduced a distraction consisting of a luminance change in the display during the gap between the offset of the central fixation ring and the appearance of the imperative stimulus. Fake-hard trials started with a cue indicating a hard trial, but were otherwise identical to easy trials and were included to allow a comparison of the effects of hard vs. easy cues on activation unconfounded by the luminance change. As this was not the goal of the present study, the analyses reported herein made no distinction between the antisaccade conditions. Fixation was the baseline condition and introduced “temporal jitter” to optimize the analysis of rapid presentation event-related fMRI data [30]–[32]. The schedule of events was determined using a technique that optimizes the statistical efficiency of event-related designs [33]. Participants performed six runs of the task lasting 5 min 16 s each and generating a total of 386 antisaccade trials and 117 fixation epochs.

Prior to scanning, participants practiced in a mock MRI scanner and were encouraged to respond as quickly and accurately as possible by making a saccade away from the stimulus. In addition to a base rate of pay, they received 5 cents for each correct response, an incentive intended to enhance attention and motivation on an otherwise boring task.

### Recording and Scoring of Eye Movement Data

The ISCAN fMRI Remote Eye Tracking Laboratory (ISCAN, Burlington, MA) recorded eye position during the scan using a 120 Hz video camera. Eye movement data were scored in MATLAB (Mathworks, Natick, MA) using a partially automated program. Saccades were identified as horizontal eye movements with velocities exceeding 47°/s. The onset of a saccade was defined as the point at which the velocity of the eye first exceeded 31°/s. Trials with initial saccades in the direction of the stimulus were scored as errors. RT was the latency of the initial saccade relative to the appearance of the stimulus.

### MRI Image Acquisition

Images were acquired with a 3.0 T Siemens Trio whole body high-speed imaging device equipped for echo planar imaging (EPI) (Siemens Medical Systems, Erlangen, Germany). Twenty-four participants were scanned with a 12-channel head coil and eleven with a 32-channel head coil. A high-resolution structural scan was acquired in the sagittal plane using 3D rf-spoiled magnetization prepared rapid gradient echo (MP-RAGE) sequences (12-channel: TR/TE/Flip = 2530 ms/3.39 ms/7°; FOV = 256 mm, 176 1.33×1×1.33 mm in-plane slices; 32-channel: TR/TE/Flip = 2530 ms/1.61+1.78*n*, *n* = 0–3/7°; iPAT GRAPPA = 3; FOV = 256 mm, 176 1×1×1 mm in-plane slices). Functional images were acquired using a gradient echo T2* weighted sequence (12-channel: TR/TE/Flip = 2000 ms/30 ms/90°, 32 contiguous horizontal slices parallel to the inter-commissural plane, voxel size: 3.1×3.1×3.7 mm, interleaved; 32-channel: TR/TE/Flip = 2000 ms/28 ms/77°, iPAT GRAPPA = 3, 41 contiguous horizontal slices parallel to the inter-commissural plane, voxel size: 3.1×3.1×3.1 mm, interleaved). The functional sequences included prospective acquisition correction (PACE) for head motion [34].

Single-shot echo planar imaging DTI was acquired using a twice refocused spin echo sequence with the same parameters for both head coils: TR/TE = 7980/84 ms; b = 700 s/mm2; 10 T2 images acquired with b = 0; 60 diffusion directions; iPAT GRAPPA = 2; 128×128 matrix; 2×2 mm in-plane resolution; 64 axial oblique (AC-PC) slices; 2 mm (0 mm gap) slice thickness; scan duration 9 min, 44 s.

### Definition of the Default and Dorsal Attention Network Regions of Interest (ROIs)

The default and the dorsal attention networks were identified using seed-based functional connectivity analyses. The seeds were defined using coordinates from prior studies [14], [35]. The default network seed was a sphere of 4 mm radius centered at the PCC (x = 0, y = −53, z = 26). The dorsal attention network seed was a combination of two 4 mm spheres centered in the intraparietal sulcus of each hemisphere (IPS; left: x = −24, y = −58, z = 52; right: x = 22, y = −58, z = 54). Pre-processing steps involved: 1) registering the motion-corrected fMRI scans to the Montreal Neurological Institute (MNI152) atlas using FSL (FMRIB Software Library, www.fmrib.ox.ac.uk/fsl); 2) spatial smoothing using a Gaussian kernel of 6 mm full-width at half maximum; 3) temporal filtering (0.009 Hz to 0.08 Hz); 4) removal of spurious or nonspecific sources of variance by regression of the following variables: (a) the six movement parameters computed by rigid body translation and rotation in preprocessing, (b) the mean whole brain signal, (c) the mean signal within the lateral ventricles, and (d) the mean signal within a deep white matter ROI. The first temporal derivatives of these regressors were included in the linear model to account for the time-shifted versions of spurious variance. Regression of each of these signals was computed simultaneously and the residual time course was retained for the correlation analysis.

Functional connectivity maps were created by computing the Pearson correlation of the signal averaged across the voxels in the seed region with that of every other voxel in the brain. Correlation maps for each individual were converted to a map of z-scores using Fisher’s z transforms. Determination of functional connectivity was based on t-tests of the z-scores at each voxel and a Bonferroni-corrected threshold based on all the voxels in the brain (p≤2*10^−7^) that set the overall probability level to p≤.05. The default network was defined as voxels with a positive correlation with the PCC seed and included the medial prefrontal cortex, angular gyrus, middle temporal gyrus and hippocampus (Table S1). The dorsal attention network was defined as voxels with a positive correlation with the IPS seed and included the frontal eye field and precentral sulcus bilaterally (Table S1). Fig. S1A shows a rendering of the default and dorsal attention networks on inflated cortical surfaces.

### fMRI Data Analysis

fMRI analyses were conducted FreeSurfer Functional Analysis Stream (FS-FAST) software. In addition to on-line motion correction (PACE), functional scans were corrected retrospectively for motion using the AFNI algorithm, normalized for intensity, and smoothed using a 3D 8 mm FWHM Gaussian kernel. Functional images were aligned to the MP-RAGE scan for each participant. Finite impulse response (FIR) estimates of the event-related hemodynamic responses were calculated for each trial position (2PRE, 1PRE, ERR, 1POST, 2POST) relative to the fixation baseline. In ambiguous cases (7.5% of trials) where a trial could be assigned to more than one type (e.g., a correct trial flanked by two errors), the trial type was picked randomly from one of the possible assignments. Hemodynamic response estimates were computed at 12 time points with an interval of 2 s (corresponding to the TR) ranging from 4 s prior to the start of a trial to 18 s after the start. Temporal correlations in the noise were accounted for by pre-whitening using a global estimate of the residual error autocorrelation function truncated at 30 s. For group-level analysis, functional images were registered to the Montreal Neurological Institute (MNI305) atlas [36] using each participant’s high-resolution structural (T1) volume.

Multiple comparison correction was based on 10,000 Monte Carlo simulations of synthesized white Gaussian noise using the smoothing, resampling, and averaging parameters of the functional analysis within the default network and dorsal attention network ROIs. This provided cluster-wise probability values (CWP) that indicate the likelihood that a cluster of a certain size would be found by chance for a given voxel-wise threshold (p≤.05).

### DTI Data Analysis

Raw diffusion data were corrected for head motion and residual eddy current distortion by registering all images to the first acquired T2 (b = 0) image, using the FLIRT tool [37] with a 12-df global affine transformation, available through the FSL software library. The diffusion tensor and FA volumes were reconstructed using the standard least-squares fit to the log diffusion signal [38]. FA volumes were registered to the high-resolution structural (T1) volumes for each participant using the T2 (b = 0) volume as an intermediary. Inter-subject registration of individual FA maps to the MNI305 atlas was performed using each participant’s T1 structural image, and the resulting transformation was applied to individual FA volumes. The MNI-normalized FA volumes were smoothed with a 3D Gaussian kernel with 6-mm full-width at half-maximum (FWHM).

### Relations of Network Activation with RT and with SATO Predictions Across Trial Positions

For each participant at each voxel we correlated activation at each of the five trial positions with the corresponding mean RT. Activation for antisaccades versus fixation at each trial position was measured at 4 s. This time point was selected based on an independent study of antisaccades showing maximum task-induced deactivation in the default network and activation the dorsal attention network at 4 s during correct trials [7]. The correlation coefficients, one for each participant, were z-transformed and a one-sample t-test was performed to determine whether, in the group, the slope of the line representing the relation between RT and activation differed from zero. For each participant, at each voxel we also computed the correlation of activation with the integer weightings representing the prediction for SATO-based adjustments at each trial position. The correlation coefficients, one for each participant, were z-transformed and subject to a one-sample t-test.

### Relations of Post-error Slowing with Network Coordination

We quantified coordination between the default (DN) and dorsal attention (DAN) networks as the interaction between network activation and trial positions 1PRE and 1POST:
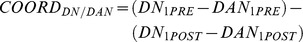



The terms in parentheses indicate mean activation at 4 s in the default and dorsal attention network ROIs for the trials preceding (1PRE) and succeeding (1POST) error trials. We related COORD_DN/DAN_ to post-error slowing using Pearson correlation. In a secondary analysis, we quantified network coordination using only voxels within each network that showed a significant (p< = .05) correlation with RT. We then computed the correlation of this more restricted index with post-error slowing. (This correlation is not circular because the correlation of task-related activation with RT quantifies this relation over trial position for each network separately while the correlation of the index of coordination with RT quantifies the relation of RT with the strength of the interaction of these networks across trial position.).

### Relations of SATO-based Changes in RT and Network Coordination to the Integrity of PCC White Matter

We defined an ROI in the posterior portion of the cingulum bundle (underlying the PCC). We identified the cingulum bundle using the Jülich histological atlas [39], as implemented in FSL and divided it into anterior and posterior segments [at y = 4 mm in MNI space; 40]. At each voxel we calculated the Pearson correlation between FA and either COORD_DN/DAN_ or post-error slowing. Multiple comparisons correction was based on 10,000 simulations of synthesized white Gaussian noise using the smoothing, resampling, and averaging parameters of the actual DTI results. Each simulated dataset was correlated with COORD_DN/DAN_ and post-error slowing. We then measured the volume of the largest cluster inside the cingulum ROI for each random dataset, resulting in a distribution of cluster volumes under the null hypothesis of no relation between the regressors and FA values, from which we estimated the significance of our actual results. We repeated these simulations in exploratory analyses of correlations of whole brain FA with COORD_DN/DAN_ and post-error slowing.

## Results

### The Effects of Trial Position on Reaction Time

Repeated measures ANOVA showed that RTs differed over the five trial positions surrounding and including the error (F(4,136) = 179.81, p<10^−6^; Fig. 1C), even when the error position was omitted from analysis (F(3,102) = 3.04, p = .03). Planned contrasts confirmed that errors were faster than correct responses (ERR<µ(2PRE, 1PRE, 1POST, 2POST; t(34) = 22.03, p<10^−6^) and that there was post-error slowing (1PRE <1POST; t(34) = 3.16, p = .003). Simple integer weightings, which represented our idealized predictions for RT across trial positions based on the SATO function (−1 for 2PRE, −2 for 1PRE, −3 for ERR, 2 for 1POST and 1 for 2POST), correlated strongly with RT (t(34) = 19.0, p<10^−10^) indicating that RT across trial position was consistent with the SATO function.

### Relations of Network Activation with RT and with SATO Predictions Across Trial Positions

We next examined whether activation during antisaccades across trial positions in the default and dorsal attention networks correlates with RT and the idealized SATO predictions. Regions of interest (ROIs) comprising the default and dorsal attention networks were defined using seed-based functional connectivity analyses (Fig. S1A). Based on the contrast of antisaccades versus fixation, regions showing task-induced deactivation overlapped with the default network ROI and regions showing task-related activation overlapped with the dorsal attention network ROI (Fig. S1B). This suggests that the anatomical components of these networks contribute to task performance.

**Figure 2:**
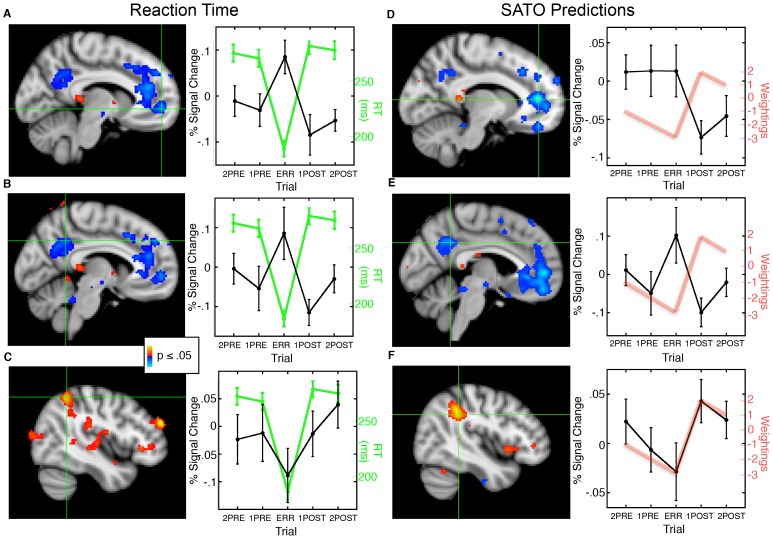
Statistical maps of the relations of task-based activation with RT (left side) and with integer weightings representing idealized predictions for SATO-based changes in RT and network activation (right side) across trial position, displayed on the MNI152 template brain. Crosshairs denote the maximum voxel in the cluster of interest. Red indicates a positive correlation and blue indicates an inverse correlation. A, B: rACC and PCC regions of the default network showed an inverse correlation with RT. Graphs plot activation at the maximum voxel (black) and mean RT (green) by trial position with standard error bars. C: The right IPS region of the dorsal attention network showed a positive correlation with RT. D-F: rACC (D) and PCC (E) regions of the default network showed an inverse correlation with prediction weightings while the right IPS region of the dorsal attention network showed a positive correlation (F). Graphs plot activation at the maximum voxel (black) and prediction weightings (red) across trial position.

Consistent with our hypothesis, activation in bilateral rostral anterior cingulate cortex (rACC) and PCC of the default network across trial positions inversely correlated with RT (Fig. 2A–B, Table 1). These regions showed greater activation in trials prior to an error, maximal activation during the error trial, and a reinstatement of task-induced deactivation after the error. The right intraparietal sulcus (IPS) of the dorsal attention network showed a positive correlation with RT and an opposite pattern of activation (Fig. 2C).

**Table 1:** fMRI activation and its relation to RT and to our theoretical prediction weightings.

Region	ClusterSize (mm^3^)	Direction	MNI Coordinates (x, y, z)	BA	Max. p-value (-base 10 log)	CWP
*Correlation with RT*						
Left rACC	2,080	r<0	−8, 52, −4	32	2.33	.02
Right rACC			2, 32, −6	24	1.86	
Left PCC	4,152	r<0	−6, −50, 36	31	2.00	.0001
Right PCC			4, −44, 28	31	1.67	
Right IPS	7,240	r>0	44. −50, 54	40	2.80	.0001
*Correlation with prediction weightings*						
Left rACC	11,088	r>0	−10, 44, 8	32	3.29	.0001
Right rACC			2, 50, 2	32	2.36	
Left PCC	2,976	r>0	−4, −50, 34	31	2.20	.001
Right IPS	5,072	r<0	44, −40, 34	40	3.03	.0001

Maxima and locations of significant clusters within the default network and dorsal attention network. Clusterwise probabilities (CWP) are based on Monte Carlo correction for multiple comparisons within the default network and dorsal attention network ROIs. Local maxima (indented) are listed for clusters spanning both hemispheres.

Because RT is influenced by a number of factors, not just error history, we also correlated activation in the default and dorsal attention networks with our idealized predictions for SATOs in RT across trial position as represented by simple integer weightings. As expected, these weightings significantly predicted activation in both the default and dorsal attention networks. Activation in the default network, specifically in bilateral rACC and PCC, was inversely correlated with the weightings (Fig. 2D–E, Table 1) and the right IPS of the dorsal attention network showed positive correlations with the weightings (Fig. 2F).

### Relations of Post-error Slowing with Network Coordination

**Figure 3:**
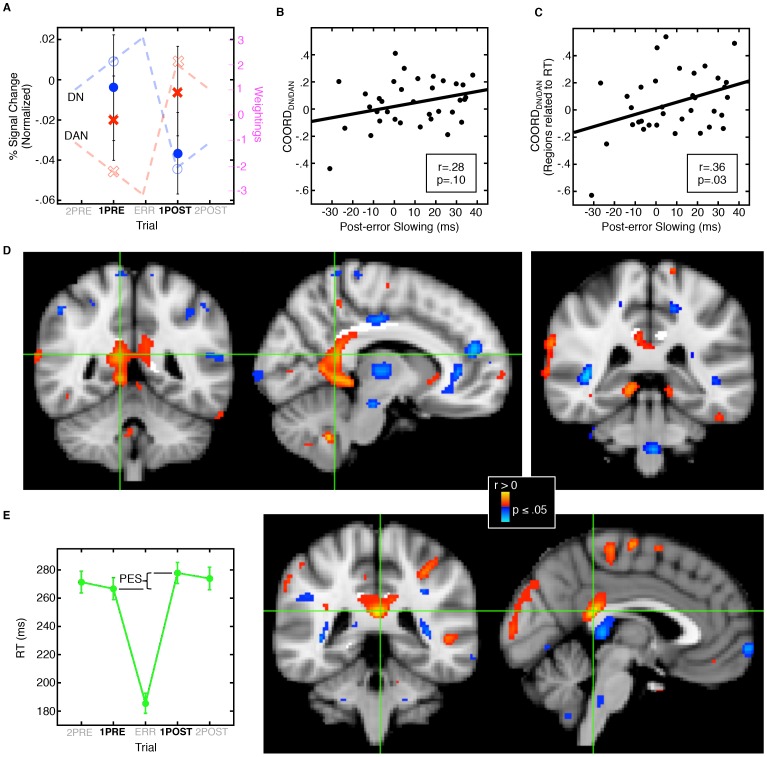
Relations of network coordination, post-error slowing, and the microstructural integrity of the posterior cingulum bundle. A: The ‘index of network coordination’ quantifies the magnitude of the reciprocal change in default relative to dorsal attention network activation from trial position 1PRE to 1POST. Dashed lines show integer weightings representing predictions for network activation across trial position, red for the dorsal attention network (DAN) and blue for the default network (DN). Open symbols (circles and x’s) are expected activation values at trials 1PRE and 1POST. Filled symbols represent means of activation with standard errors at these positions. For display purposes only, activation values were normalized based on the mean activation across all five trial positions. B: Correlation of network coordination (COORD_DN/DAN_), and post-error slowing. C: Same correlation as B, but with the definition of COORD_DN/DAN_ restricted to network regions in which activation correlated with RT (see Fig. 2A–C). D: Statistical maps of correlations of FA with COORD_DN/DAN_ displayed on the MNI152 template brain. The posterior cingulum bundle ROI is highlighted in white. Warm and cool colors indicate positive and negative correlations, respectively. Crosshairs in the left and middle images show coronal and sagittal views of the voxel of maximal significance (MNI coordinates: 10, −48, 16). The right image shows that the region of correlation extends along the cingulum bundle to its termination in the parahippocampal gyrus. E: Plot of mean RT across trial position with standard error bars (left) indicating the measurement of post-error slowing (PES). PES was defined as the difference in RT between 1POST and 1PRE. Statistical maps of correlations of FA with post-error slowing (right; peak MNI coordinates: −2, −40, 20).

Post-error slowing, defined as the difference in RT between the correct trials immediately following (1POST) and preceding (1PRE) an error, is a highly reliable and widely reported index of the SATO. We expected that post-error slowing would be paralleled by reciprocal changes in activation in the default and dorsal attention networks. We quantified these network changes as the interaction between network activation in trial positions 1PRE and 1POST (Fig. 3A). A higher value indicates relatively high default network and low dorsal attention network activation prior to the error and a reversal of that pattern following the error. This ‘index of coordination’ between the default (DN) and dorsal attention (DAN) networks (COORD_DN/DAN_) correlated with post-error slowing at a trend level (r(33) = .28, p = .10; Fig. 3B). When we restricted COORD_DN/DAN_ to regions within each network that correlated with RT (Fig. 2A–C), the relation with post-error slowing reached significance (r(33) = .36, p = .03, Fig. 3C). These findings suggest that the magnitude of post-error slowing in individual participants is influenced by the degree of reciprocity between the networks.

### Relations of Network Coordination, Post-error Slowing, and the Microstructural Integrity of the Posterior Cingulum Bundle

To test the hypothesis that the network coordination underlying the SATO relies on the structural connections of the PCC, we correlated FA in the posterior cingulum bundle with both the index of network coordination ‘COORD_DN/DAN_’ and post-error slowing. Increased FA in the posterior cingulum bundle was associated with greater network coordination and this relation reached significance in the right cingulum ROI (peak MNI coordinates: 10, 48, 16, cluster size: 784 mm^3^, clusterwise probability: 0.04; Fig. 3D). Similar correlations were seen with post-error slowing (Peak coordinates: −2, −40, 20; Fig. 3E), but failed to reach significance (CWP: 0.18 in right and left ROIs). The regions of correlation followed the course of the cingulum bundle around the splenium of the corpus callosum into the parahippocampal gyrus. No clusters in the whole brain analyses significantly correlated with COORD_DN/DAN_ or post-error slowing.

## Discussion

Our findings are consistent with the hypothesis that the balance of activity between the default and dorsal attention networks underlies SATOs in response to behavioral outcomes and is mediated by the PCC. First, we found that the pattern of RT over a series of trials surrounding and including an error was consistent with predictions based on an idealized SATO function. Second, over this series of trials, RT correlated with activation in the default and dorsal attention networks, but in opposite directions. In the default network, bilateral rACC and PCC showed greater activation in trials prior to and including an error and this corresponded with faster responses, presumably reflecting a focus on the internal milieu at the expense of attention to the task that culminated in an error. In the trial following an error, responses slowed and task-induced deactivation was reinstated, suggesting a reduction in internally-directed attention. In the dorsal attention network, activation in the right IPS correlated positively with RT. The right IPS is thought to be dominant in directing attention and eye gaze to relevant parts of extrapersonal space [41]. This region showed reduced activation up to and including the error, and increased activation after the error, possibly reflecting renewed attention to the task. Finally, greater integrity of the posterior cingulum bundle was associated with greater network coordination as indexed by the magnitude of reciprocal changes in activation between the two networks in the trials immediately before and after an error. These findings support the theory that error-based SATOs in RT are mediated by dynamic changes in the allocation of attention to the internal and external milieu, and that these changes are mediated by the PCC.

Our results are consistent with prior demonstrations of increased default network activation prior to and during errors [7], [16] followed by decreased default network activation and a corresponding increase in dorsal attention network activation after errors [8]. The present study adds to this literature by demonstrating this pattern within individual participants using a series of trials of the same task and by correlating changes in activation with both RT adjustments and the microstructural integrity of the posterior cingulum bundle. Previous work suggests that the PCC is a cortical hub [19]–[21] that detects errors and signals the need for immediate correction [24]. The present findings suggest that in response to errors the PCC also mediates shifts in task engagement to optimize behavioral outcomes. Specifically, increased FA of the posterior cingulum bundle predicted more pronounced reversals in the polarities of default and dorsal attention network activation following an error, and this correlated with more pronounced post-error slowing. These relations were strongest near the border of the ventral and dorsal divisions of the PCC, defined as the ventral branch of the splenial sulci [42]. This area is thought to regulate interactions between the default and dorsal attention networks [22], [23]. In this context, the relations of the integrity of the white matter underlying this region with reciprocal changes in network activation and error-based adjustments to RT suggest that more efficient connections of the PCC lead to stronger dynamic modulation of network activation and behavior in response to errors.

Our findings contrast with a prior report that task-induced deactivation of the default network is associated with faster, rather than slower responses [18]. This apparent discrepancy likely reflects differing task requirements. In the selective attention task of the prior study greater engagement of attention would presumably lead to faster responding. For antisaccades and other tasks requiring response inhibition, however, greater engagement following an error corresponds to greater inhibitory control and, consequently, longer RTs. (This is consistent with single unit recordings in monkeys that show a relation between the inhibition of saccade-related neurons in the frontal eye field and slower, more accurate responding [28]). Both studies support an association between default network activation and fluctuations in RTs indicative of task engagement. The present findings further suggest that the relations between default and dorsal attention network activation underlie these adjustments.

Prior studies of the neural basis of SATOs have focused on the effects of instructions that emphasize the importance of speed over accuracy or *vice versa* [43]. Computational models suggest that changing instructions alters the threshold for triggering a response [44]–[46]. When speed is emphasized, less evidence is required to trigger a response, and this is paralleled by activation changes in task-relevant regions [44]–[50]. These cue-mediated SATOs may differ from SATOs in response to behavioral outcomes during which instructions remain constant. Cue-mediated SATOs are thought to reflect strategic changes in decision-making to focus either on responding more quickly with a minimum of evidence or more deliberately once enough evidence has accumulated. In this situation, attention may remain relatively constant and may not be a factor in mediating the observed SATOs in RT. In contrast, errors are associated with changes in attention [6] and SATOs in response to aversive and unexpected outcomes such as errors may reflect increased attention that prompts slower and more careful responding. While we interpret the adjustments in RT and corresponding changes in network activation around errors to reflect alterations in attention and task engagement, this does not exclude the possibility of a corresponding change in evidence-based response thresholds. If this were the case, however, we might have expected to see RT correlations in the frontal eye field, which is also part of the dorsal attention network and is the key cortical region for triggering volitional saccades [51]. Instead, we observed correlations with RT in the right IPS, which is dominant in spatially-directed attention [41]. This, along with the pattern of network activation changes around an error, leads us to interpret our results as reflecting fluctuations in attention and task-engagement in response to behavioral outcomes, and to link these fluctuations to intrinsic brain networks, and to mediation by the PCC.

The evidence that SATOs in RT are mediated by shifts of attention can be reconciled with current theory that SATOs reflect alterations in response thresholds by recognizing that SATOs in RT arise under different circumstances and that motor responses are unlikely to be governed by a single process. For example, during antisaccades, as task-directed attention wanes (i.e., on trials leading to an error), there may be a corresponding relaxation of the response threshold, which allows faster responding, until a ‘slip’ occurs. After the error, participants may increase attention and adopt a stricter threshold (i.e., require more evidence), leading to slower responses. Changes in RT, and presumably attention and response threshold, are paralleled by changes in the activity in regions of the dorsal attention network that are activated by the task (see Fig. S1). The proposal that changes in both attention and response thresholds modulate SATOs is consistent with recent evidence from monkey single neuron recordings from the frontal eye field during performance of a saccadic task [52]. Cues that prompted SATOs modulated the activity of neurons representing the ‘salience’ of visual information in addition to those involved in generating the response. This suggests that SATOs reflect adjustments of both attention and motor response thresholds.

In summary, the present findings add to the literature on SATOs in RT around errors by showing that they are accompanied by reciprocal changes in activation of the default and dorsal attention networks. Our findings suggest that dynamic changes in attention to the internal versus external milieu in response to errors underlie error-based SATOs in RT and are mediated by the PCC. This ability to rapidly and dynamically modulate task engagement in response to behavioral outcomes is fundamental to adaptive, flexible responding.

## Supporting Information

Figure S1 **Default and dorsal attention network regions of interest and task-related activation.** Volume-based statistical maps were projected onto the inflated cortical surfaces of a template brain for visualization. Gray masks cover subcortical regions in which activity is displaced. A: Default (blue) and dorsal attention (red) network regions of interest (ROIs). B: Task-related activation for the contrast of all correct trials vs. fixation at 4 s. Warm colors indicate stronger activation during the task than during fixation, and cool colors indicate stronger activation during fixation.
(TIF)

Table S1 **Regions comprising the default and dorsal attention network ROIs.** List of maxima and locations of clusters showing significant positive functional connectivity with the seed region for each network in the group data. Only clusters larger than 1000 mm^3^ are reported.
(DOCX)
