# Correction: Network Dynamics Underlying Speed-Accuracy Trade-Offs in Response to Errors

**DOI:** 10.1371/annotation/5db627f0-4ac8-4d17-827d-a8fef1552b81

**Published:** 2013-11-05

**Authors:** Yigal Agam, Caitlin Carey, Jason J. S. Barton, Kara A. Dyckman, Adrian K. C. Lee, Mark Vangel, Dara S. Manoach

This article was republished on October 31, 2013 because of improperly cropped figures. This republication addresses the errors noted in the previous comments. Please download this article again to view the full figures. 

