# Supplementary material for: Network Dynamics Underlying Speed-Accuracy Trade-Offs in Response to Errors
Source: PLoS One. 2013 Sep 12;8(9):e73692. doi: 10.1371/journal.pone.0073692 (PMC3772006; doi:10.1371/journal.pone.0073692)
Supplement: Table S1 — Regions comprising the default and dorsal attention network ROIs. List of maxima and locations of clusters showing significant positive functional connectivity with the seed region for each network in the group data. Only clusters larger than 1000 mm3 are reported. (DOCX) [file pone.0073692.s002.docx]

Table S1: Regions comprising the default and dorsal attention network ROIs. List of maxima and locations of clusters showing significant positive functional connectivity with the seed region for each network in the group data. Only clusters larger than 1000 mm^3^ are reported.

| Region | Cluster Size (mm^3^) | MNI Coordinates | | | Brodmann Area | Max. p-  value (-base 10 log) |
| --- | --- | --- | --- | --- | --- | --- |
|  |  |  |  |  |  |  |
|  |  | x | y | z |  |  |
| *Default Network* | | | | | | |
| PCC | 37392 | 0 | -54 | 26 | 31 | 18.3 |
| rACC | 38584 | 0 | 50 | -8 | 32 | 14.3 |
| Left angular gyrus | 4008 | -50 | -70 | 32 | 39 | 12.2 |
| Right angular gyrus | 8528 | 46 | -68 | 38 | 39 | 11.8 |
| Left inferior temporal gyrus | 2792 | -58 | -12 | -22 | 21 | 11.4 |
| Right middle temporal gyrus | 3112 | 60 | -10 | -22 | 21 | 10.3 |
| Right parahippocampal gyrus | 1656 | 24 | -36 | -16 | 36 | 9.6 |
| *Dorsal Attention Network* | | | | | | |
| Left IPS (extending bilaterally) | 97008 | -22 | -60 | 52 | 7 | 17.4 |
| Left precentral sulcus | 2008 | -40 | -4 | 40 | 6 | 10.7 |
| Right precentral sulcus | 1200 | 50 | 2 | 36 | 6 | 10.0 |
| Right FEF | 2160 | 28 | 0 | 60 | 6 | 9.9 |
| Left FEF | 3368 | -24 | 0 | 60 | 6 | 9.6 |
